# Circulating Tumor Cell Detection in Lung Cancer: But to What End?

**DOI:** 10.3390/cancers11020262

**Published:** 2019-02-23

**Authors:** Véronique Hofman, Simon Heeke, Charles-Hugo Marquette, Marius Ilié, Paul Hofman

**Affiliations:** 1 Laboratory of Clinical and Experimental Pathology, CHU Nice, FHU OncoAge, University Côte d’Azur, 06100 Nice, France; hofman.v@chu-nice.fr (V.H.); heeke.s@chu-nice.fr (S.H.); ilie.m@chu-nice.fr (M.I.); 2 Team 4, IRCAN, FHU OncoAge, University Côte d’Azur, CNRS, INSERM, 06107 Nice CEDEX 02, France; marquette.c@chu-nice.fr; 3 Hospital-Integrated Biobank (BB-0033-00025), CHU Nice, FHU OncoAge, University Côte d’Azur, 06100 Nice, France; 4 Department of Pneumology and Oncology, CHU Nice, FHU OncoAge, University Côte d’Azur, 06100 Nice, France

**Keywords:** circulating tumor cells, liquid biopsy, lung cancer, personal medicine, techniques, xenograft

## Abstract

The understanding of the natural history and biology of lung cancer has been enhanced by studies into circulating tumor cells (CTCs). Fundamental and translational research, as well as clinical trials in the characterization and behavior of these cells, have constantly contributed to improving understanding within the domain of thoracic oncology. However, the use of these CTCs as prognostic and predictive biomarkers has not been adopted to the same extent as circulating free DNA (cf-DNA) in plasma, in the daily practice of thoracic oncologists. However, recent technological advances have firmly put the detection and characterization of CTCs in thoracic oncology back on the agenda, and have opened up perspectives for their routine clinical use. This review discusses the major advances of using CTCs in the domain of thoracic oncology, as well as the envisaged short- and long-term prospects.

## 1. Introduction

Liquid biopsy (LB) plays a major role in thoracic oncology [1,2]. A number of recent publications and developments within this domain testify to the increasing importance of LB. These studies concern not only fundamental translational and clinical research, but also technological advances [3,4,5,6,7]. They have provided a better understanding of the molecular and cellular mechanisms, the progression of lung cancer, and the treatment of patients. Among these studies, research into mutations in *EGFR* using plasma circulating free DNA (cf-DNA) have led to the use of LB in the clinical routine for patients with advanced stage or metastatic non-small cell lung carcinoma (NSCLC) [2,8,9,10]. This approach is now used in a large number of hospitals.

The number of detectable biological targets in an LB that are potentially accessible to treatment has increased, and future application of different biomarkers can be envisaged in the short-term [11]. The complexity of molecules for detection in the blood of patients with lung cancer has increased with advances in our understanding of the biology of the different components circulating in the blood. These components include free or complexed nucleic acids, microparticles including exosomes, circulating “non-hematological” cells including circulating tumor cells (CTCs), and proteins of serum and plasma [12,13,14,15,16]. The addition to these analyses of different circulating hematological normal cells (neutrophils, lymphocytes, monocytes, platelets), constituting a “liquid microenvironment”, has progressively been envisaged [17,18].

While taking into account the increasing complexity, a number of biomarkers have been developed for use, particularly in the clinic, for the interests of patients with advanced or metastatic lung cancer. Thus, the possibility of detecting activating or resistance mutations induced by molecular therapeutics in plasma cf-DNA has been associated with an explosion in the number of exploratory methods and applications in thoracic oncology [2,19,20,21,22]. One of the consequences of these rapid developments concerns the progressive decrease in the interest shown in the analysis of CTCs in thoracic oncology, at least for routine daily practice [23]. However, cf-DNA and CTCs are complementary, and can serve to answer different questions [24]. While genetic assessment might be suitable with both cf-DNA and CTCs, only CTCs might be able to give insights into the seeding of metastases and interactions of CTCs with other circulating blood cells, endothelial cells and, subsequently, different parenchyma [25,26]. cf-DNA and CTCs can be successfully simultaneously assessed in the same patient for a broader insight of tumor burden [27,28,29]. The absence of robust approaches for the detection of CTCs in clinical routine practice, in the context of the healthcare of these patients, probably explains the decline in interest. This is also due to the facts that CTCs are rarely found in blood, for capture, and that the capturing techniques, which are both very sensitive and specific, still require validation to provide optimal results for use in daily practice [30,31]. A selection of key studies on CTC isolation techniques have been summarized in Table 1. In this regard, the fact that different methods of CTC isolation give conflicting results for the same series of patients has certainly slowed the interest shown in this domain by many investigators [32,33]. Fewer groups around the world study CTC detection compared to groups working on detection of cf-DNA in the area of thoracic oncology. A number of review articles have discussed the advantages and limits of using CTCs or plasma cf-DNA in oncology [34,35,36,37]. The majority underline the difficulty of using CTCs as prognostic and predictive biomarkers in daily practice. Where, then, lies the interest in—and the role of—projects aimed at detecting and characterizing CTCs in thoracic oncology? Is it possible to envisage, in the future, the routine use of this type of analysis in the clinic?

This review aims to outline how the study of CTCs in an LB can provide unique and indispensable information in thoracic oncology, and to present the future long- and short-term developments in this domain.

## 2. Opportunities Offered by Studying CTCs in Thoracic Oncology

A couple of opportunities can be specifically associated with CTC detection programs only (Table 2).

### 2.1. Developing Xenografts from Circulating Tumor Cells and Cells Cultured In Vitro

**Figure 1:**
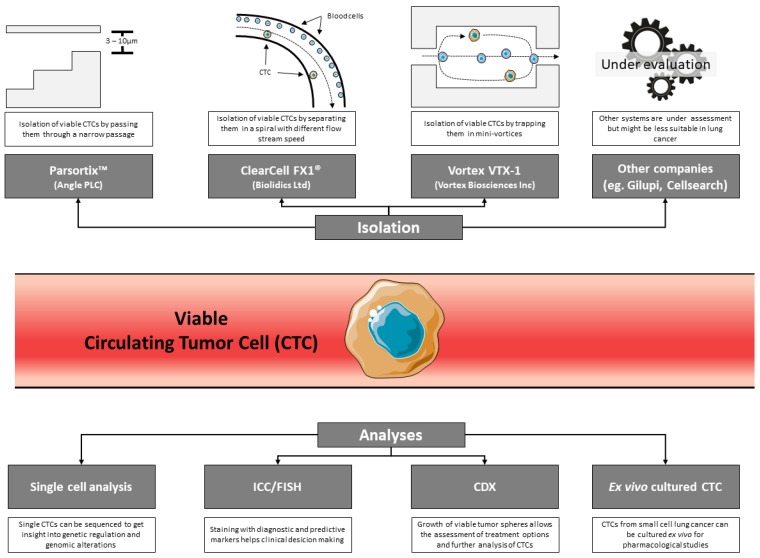
Overview of the different isolation techniques and possibilities in CTC research. Different devices have been developed with Parsortix (Angle PLC, Guildford, UK), ClearCell FX1 (Biolidics, Singapore), and Vortex VTX-1 (Vortex Biosciences, Pleasanton, CA, USA) being the most prominent. However, other CTC isolation systems, like GILUPI (Potsdam, Germany), can also be used for the isolation of viable CTCs. Isolation of viable CTCs then allows for the processing and analysis of cells using numerous approaches.

Different enrichment techniques allow for the isolation of “viable” CTCs from patients with lung cancer (ClearCell^®^ FX System, VTX-1 Liquid Biopsy System, Parsortix™ Cell Separation System) [72,73,74] (Figure 1). These techniques represent a crucial development in the use of LB in thoracic oncology. By injecting CTCs into mice, CTC-derived xenograft (CDX) models can be obtained, and the biology of these cells can be studied in vivo. This approach allows for analysis of the proliferation and level of “aggressiveness” of CTCs, their behavior once extravasated from blood and, thus, their metastatic potential. CDX can be developed to examine the response of tumors to different therapeutic molecules and protocols. In theory, these studies can anticipate the response of tumors to certain treatments, depending on the patient, and thus allow the most effective treatment to be proposed. Additionally, CDX can also be used to study primary and secondary mechanisms of resistance to therapeutic molecules. However, the setup of the methodology of CDX is difficult, and the systems of cell enrichment for the isolation of CTCs show variable sensitivity (ClearCell^®^ FX System, VTX-1 Liquid Biopsy System, Parsortix™ Cell Separation System) [72,73,74]. Finally, the rate of successful development of CDX depends on the number of cells isolated and their ability to proliferate.

At present, the development of CDX in thoracic oncology concerns predominantly small cell lung carcinomas (SCLCs), as shown by several publications in this domain on this type of histology [51,52,53,75]. One reason for this is the high number of CTCs in the blood of patients with SCLC when at a metastatic phase (mean ± SD = 1589 ± 5565 in 7.5 mL of blood), and another is due to the capacity of these CTCs to proliferate [59]. Using these model systems, it is possible to envisage treating patients according to the response of the xenografts to different tested molecules [54,76,77,78]. Complementary analyses can be performed using cells cultured after dissociation of the CDX tumor [54]. This method allows several million tumor cells to be cultured and tested with different therapeutic molecules [54]. By contrast, few studies concern CDX obtained from NSCLC. Fewer cells are isolated in NSCLC, and their ability to proliferate is lower in comparison to SCLC. Methods have been developed to culture, in vitro, the CTCs, and to thus to analyze their potential to proliferate, their biology, and their sensitivity to different molecules [79]. These approaches are not as advanced as CDX for clinical application.

### 2.2. Single-Cell Analysis and Functional Studies

In contrast to studies on circulating nucleic acids, exosomes, or other blood biomarkers, the analysis of CTCs can define their molecular genetics, epigenetics, transcriptomics, and protein profile [55,56,58]. Thus, a very precise tumor profile and characterization of the phenotype of cells with invasive potential can be studied and can contribute to analyses concerning tumor heterogeneity [30]. Single-cell comparative analyses can be performed with primitive tumors, CTCs, and metastatic tumors from the same patient and thereby provide complementary information concerning the biological mechanisms associated with the progression and dissemination of lung cancers. Using isolated or cultured live cells, these functional studies identify the proteins secreted by CTCs [55]. The EPISPOT technology applied to these live isolated cells is particularly sensitive for the study of the expression and secretion of proteins by CTCs [55]. It has also been demonstrated that CTCs might be directly cultured on microfilters that are used for CTC isolation. This might facilitate CTC culture as it avoids the complicated transfer of CTCs onto cell culture plates, and might strongly increase the time from CTC isolation to plating in a culture medium for growth [80].

Additionally, the development of new technologies, such as the DEPArray^TM^ (Menarini Silicon Biosystems, Bologna, Italy), allows the separation of single cells from a pool of isolated CTCs to get further insight into single-cell dynamics [81,82]. This has been used to determine copy number variations (CNVs) in SCLC patients upon single-cell DNA sequencing [39]. Additionally, NanoArrays have been developed for the single-cell analysis of NSCLC [83].

Finally, single-cell RNAseq approaches from CTCs have been successfully implemented for different solid tumors, like breast and prostatic carcinoma [84,85], and should probably be in use also for lung cancer in the near future [86].

### 2.3. Correlation between Cytopathological and Molecular Phenotypic Analyses

Some methods of detection of CTCs can visualize and identify the classical cytological criteria of cancer cells that are routinely used in the laboratory for different cytological samples [60,61,87]. The identification of different diagnostic biomarkers (TTF1, p40) or of the predictive response to a therapeutic (ALK, ROS1, PD-L1) can be correlated to these cytomorphological criteria, which considerably increase the specificity and reliability of these methodological approaches [42,62,63,64,65,66,67,87]. Several studies have reported the extensive morphological heterogeneity of NSCLC CTCs. All the cytological criteria of malignant cells, as well as other criteria and circulating “non-hematological” cells without cytonuclear anomalies were identified. While considering the latter cells, the following questions can be raised: Are they tumor cells? Are they cells with an invasive potential? Or are they normal epithelial cells associated, or not, with CTCs [58,60,87]?

It has been therefore proposed to further categorize CTC in different classes, like disseminated tumor cells (DTC), CTCs undergoing epithelial-to-mesenchymal transition (EMT) (EMTCTCs), and cancer-associated macrophage-like cells (CAMLs), which will further challenge the precise detection and characterization of CTCs [43,88,89,90,91].

While the heterogeneity is challenging for the successful identification of CTCs, it also has strong implications on the prognosis, as especially CTCs with a mesenchymal phenotype might have a more severe impact on spreading disease than CTCs with an epithelial phenotype [92,93,94].

## 3. What Are the Prospects?

One of the main hurdles facing the analysis of CTCs concerns the large number of methods that have been developed to isolate and characterize CTCs. The number of techniques makes it difficult for an operator to understand and choose a technique, particularly for routine clinical use. The selection by an investigator of a technique is guided by several parameters: (i) the sensitivity and specificity, (ii) the ease of use, (iii) a rapid turnaround time for getting results, (iv) the reproducibility, and (v) the cost. To date, no method answers all these parameters to analyze CTCs in daily practice in the domain of thoracic oncology. Hence, some one concern is improving the methods of detection and characterization of CTCs to make them as competitive as the detection and characterization of cf-DNA, which has been widely adopted in routine clinical practice. Since CTCs in blood are rare occurrences (particularly in NSCLC patients), the optimization of CTC enrichment is essential. Progress in this area will be achieved through a better understanding of the biology of CTCs and the discovery of new specific biomarkers of CTCs, in particular, if they identify “viable” CTCs with aggressive and metastatic potential.

A new avenue of biological investigation has recently emerged with the study of active interactions between circulating hematological cells and CTCs [68,95]. These studies should lead to the discovery of new mechanisms of resistance to cell death by CTCs and, thus, to novel therapeutic targets that induce cell death [68,95].

As for the analyses performed with cf-DNA in plasma (analysis of mutations or of methylation), or other blood biomarkers (plasma microRNA, auto-antibodies, fragments of complement, and plasma proteins), some recent studies suggest that CTCs may be early markers of lung cancer, which may even be detected several months before radiographic emergence of the cancer [69,70,71,96]. However, these approaches require (i) optimization of the cellular enrichment methods and characterization, (ii) several independent studies, and (iii) inclusion of a large number of patients for validation.

Single-cell genetic and transcriptomic analyses must provide new information on very specific molecular targets for novel therapeutics to be used in the context of personal medicine. Recently developed technological approaches that are being evaluated may contribute to better characterization of CTCs at the single-cell level [58,97]. These complex methodological developments should allow for better understanding of the heterogeneity of CTCs among patients and for the same patient, as well as for identification of CTCs from either primary or metastatic tumors. The molecular characterization of CTCs may compete with or, more likely, provide important complementary information to that obtained from circulating free nucleic acids. One of the obstacles lies in the heterogeneity of CTCs and the variable expression of molecular markers depending on the cell (or cells) isolated [30,39].

Several studies indicate that comparison of the quantity of patient CTCs—at baseline and after treatment—may be a good indicator of prognosis in SCLC [51,98]. Thus, for this pathology, aside from the quantification of cf-DNA in plasma or, as reported more recently, of the tumor mutation load, CTCs may be used, in routine practice as an indicator of the response of tumors to treatment. The possibility of establishing CDX or cells in culture originating from CTCs, and thus of testing therapeutic molecules ex vivo, may benefit the clinical follow-up and care of patients [54,77,78,98]. In a similar manner, cell cultures of millions of cells obtained from CDX should facilitate their molecular analysis [54]. One of the limitations of this approach is the time required to obtain CDX, which is not compatible with care of the majority of patients from whom the CTCs were obtained.

Despite the number of promising studies on NSCLC, the use of CTCs in routine practice remains hypothetical, in particular, for use as a prognostic biomarker. In fact, the quantification of CTCs in this pathology strongly varies according to the techniques used for the same patient, which makes this approach ineffective [33,38,45,99,100,101,102]. Moreover, the establishment of CDX is rather challenging for NSCLC [102].

New techniques for detection and characterization of CTCs need to be continually evaluated and examined, which may be difficult for an individual cohort of patients. The standardization of protocols for the isolation, preparation, enrichment, and characterization of CTCs is a prerequisite to presenting international ISO (International Organization for Standardization) norms before their routine clinical use by thoracic oncologists.

## 4. Conclusions

In thoracic oncology, the use of CTCs is often associated with issues concerning translational research that does not involve immediate use in routine practice. Thus, projects developed with CTCs contrast with applications using plasma cf-DNA that have been employed for a number of years for the care of patients, in particular, in the detection of activating mutations or mutations conferring resistance in *EGFR* [2,8,9,10]. Until now, a number of technological hurdles prevented the transfer of applications using CTCs into daily practice in thoracic oncology. Transfer to routine practice in real life can only be achieved if solid benefit to the patients is demonstrated, such as choice of therapy according to the number and type of CTCs as well as the expression of certain biomarkers of interest and, more importantly, real benefit in terms of overall survival of patients.

Technological progress on the analysis of CTCs should lead not only to the discovery of novel molecular targets for early diagnosis, but also to new prognostic and predictive biomarkers of the response or resistance to therapeutics. One promising direction concerns the development of CDX, allowing the expansion of tumor cells and their analysis in vivo, as well as the possibility of testing new therapeutic strategies. However, the success of CDX depends on the number of isolated CTCs, which is very low for certain histological types of lung cancer. 

The combined and simultaneous study of several elements of LB (CTCs, free circulating nucleic acids, exosomes, proteins, etc.) may permit better assessment of the different phenotypes found using LB, and the associated individual approaches to define reliable diagnostic, prognostic, and predictive parameters [103,104]. Technological progress will permit the combination of different biomarkers at the single-cell level, and will increase our knowledge of CTCs [105,106]. A continual increase in different biomarkers for studying individual patients will evolve from more and more complex studies. In addition, as recently emerging in other areas of medicine, artificial intelligence should rapidly emerge and could integrate the different elements of LB, including CTCs [107,108]. Consequently, CTCs certainly play a key role in this context but, operationally, require further development before coming into daily routine use in thoracic oncology.

## Figures and Tables

**Table 1:** Technical advancements in circulating tumor cell (CTC) research for lung cancer.

Study	Histology	Approach	Method	Results	Ref
Hofman et al.	NSCLC	Analysis of preoperative CTCs to predict relapse in early stage NSCLC patients.	ISET™ (Rarecells, Paris, France)	Circulating non-hematologic cells were detected in 102/208 patients with patients with >50 cells having worse prognosis	[38]
Hofman et al.	NSCLC	Assessment of CTCs before radical surgery as prognostic factor.	ISET (Rarecells) and CellSearch™ (Menarini Silicon Biosystems, Bologna, Italy)	CTCs were detected in 69% (144/210) of patients but only in 20% (42/210) of patients with both ISET and CellSearch. Patients where CTCs were detected with both methods had worse prognosis	[33]
Carter et al.	SCLC	Assessment of copy number alterations in CTCs to distinguish chemosensitive from chemorefractory patients	CellSearch™ (Menarini Silicon Biosystems)	31 patients tested. 27–20,815 CTCs per 7.5 mL of blood (median, 836). 83.3% correctly classified cases	[39]
Drapkin et al.	SCLC	Generation of CTC-derived Xenografts.	CTC-iChip^neg^ device ^‡^	CDX could be obtained with an efficiency of 38%	[40]
Tan et al.	NSCLC	Comparison of EML4-ALK FISH in CTCs and tumor tissues	ClearCell FX™ (ClearBridge Biomedics, Singapore, Singapore)	>90% of concordance. More CTCs in EML4-ALK positive patients (3–15/1.88 mL blood) than in negative patients (0–2).	[41]
Ilie et al.	NSCLC	Analysis of PD-L1 expression on CTCs and white blood cells compared to tumor tissue.	ISET™ (Rarecells)	PD-L1 in CTCs can be detected at 93% concordance to tumor tissue and 73% in white blood cells	[42]
Adams et al.	NSCLC	Sequential analysis of PD-L1 and RAD50 expression in patient undergoing radiotherapy.	CellSieve™ (Creatv MicroTech, Rockville, MD, USA)	CTCs and cancer-associated macrophage-like cells (CAMLs) were detected in up to 100% of 41 patients and presence increased during treatment. RAD50 and PD-L1 expression also increased over time	[43]
Chudziak et al.	SCLC	Comparison of Parsortix™ and CellSearch™ devices for clinical evaluation.	Parsortix™ (Angle PLC. Guildford, UK) and CellSearch™ (Menarini Silicon Biosystems)	1–3780 CTCs per 7.5 mL of blood in CellSearch™ (10/12 samples) and 20–1474 using Parsortix™ (12/12 patients)	[44]
Krebs et al.	NSCLC	Comparison of ISET™ with CellSearch™	ISET (Rarecells) and CellSearch™ (Menarini Silicon Biosystems)	80% positive patients using ISET™ (0–1045, mean = 71 cells) compared to 23% in CellSearch™ (0–78, mean = 4 cells)	[45]
Gorges et al.	NSCLC	Comparison of CellSearch™ with GILUPI CellCollector™	GILUPI CellCollector™ (GILUPI, Potsdam, Germany) and CellSearch™ (Menarini Silicon Biosystems)	58% positive patients with GILUPI™ (1–56, median = 5 cells) compared to 27% with CellSearch™ (1–300 cells)	[46]

^‡^ The CTC-iChip^neg^ is not commercially available. Abbreviations: non-small cell lung carcinoma (NSCLC), small cell lung carcinoma (SCLC).

**Table 2:** Methodological approaches in CTC research and main issues.

Approaches	Interests	Issues	Ref
CTCs cultured ex vivo	Drug testing Genomic/transcriptomic profiling Assessment of metastatic cells	Depends on the number of viable isolated cells Lack of microenvironment	[47,48,49,50]
CDX	Drug testing Genomic/transcriptomic profiling	Lack of human immune cells in microenvironment Long duration to obtain xenograft	[40,51,52,53]
CTC-derived explant	Expanding tumor-derived cells Large potential for drug screening	Lack of microenvironment Long duration to establish	[54]
Single-cell analyses	Genomic/transcriptomic profiling Tumor heterogeneity studies Functional studies (secretion)	Difficult to get isolated viable CTCs Technologically challenging	[55,56,57,58]
Microemboli tumor cells	Impact on prognosis Cell–cell contact interaction studies Heterogeneity studies	Difficulty to separate the different CTCs from a cluster	[59]
CTCs & circulating immune cells interaction	Mechanisms of crosstalk between cells	Different populations of immune cells Lack of ex vivo models	[17]
Cytomorphological assessment	Identification and characterization of specific populations of interest In situ protein and RNA assessment linking to the cell morphology	Highly dependent on the isolation technique	[42,60,61,62,63,64,65,66,67,68]
CTCs quantification at baseline and monitoring	Real time monitoring of systemic anticancer therapies	No FDA approved test for lung cancer	[69,70,71]

CDX = CTC-derived xenograft.
